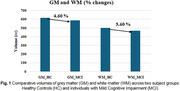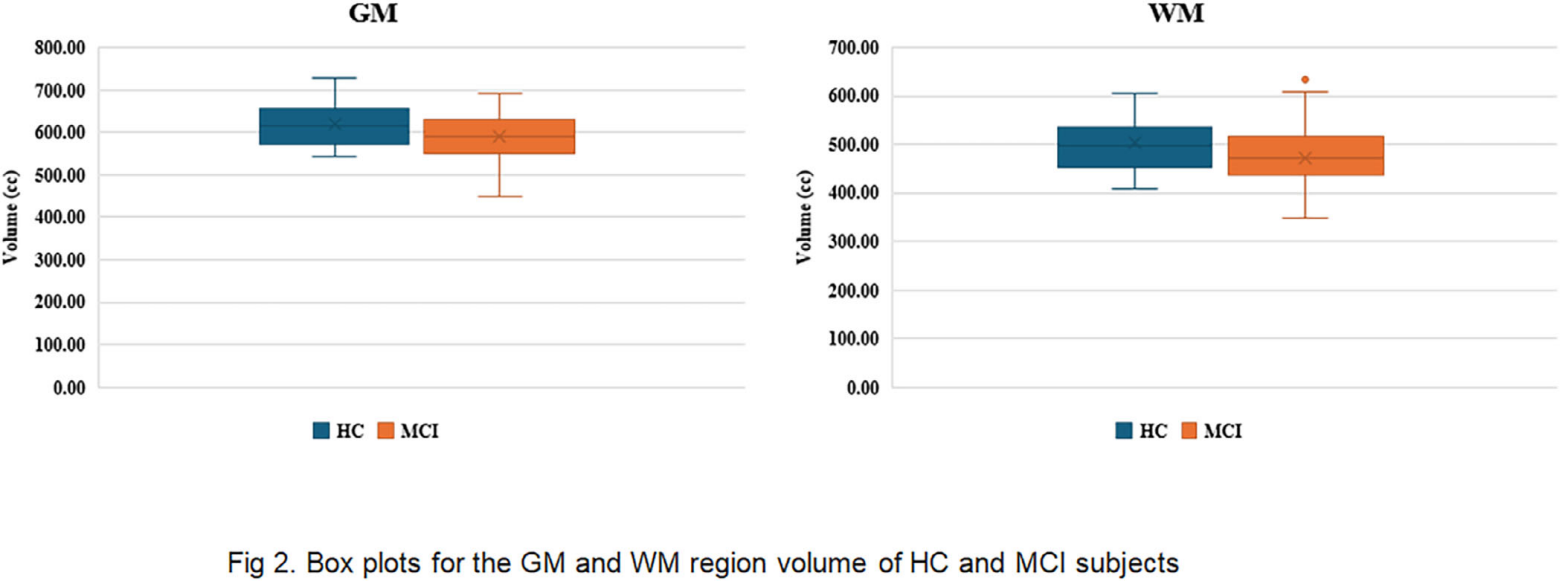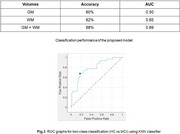# Quantitative analysis of volumetric changes in gray and white brain matter in mild cognitive impairment

**DOI:** 10.1002/alz70856_106355

**Published:** 2026-01-08

**Authors:** Marufjon Salokhiddinov, Dileep Kumar, Dharmesh Singh, Akash Gandhamal, Rustam Fayzullaev

**Affiliations:** ^1^ Tashkent Medical Academy, Tashkent, Tashkent, Uzbekistan; ^2^ Central Research Institute, Shanghai, Shanghai, China; ^3^ Cenral Research Institute, Delhi, Delhi, India; ^4^ National Olympic Committee, Tashkent, Tashkent, Uzbekistan

## Abstract

**Background:**

Mild cognitive impairment (MCI) represents a transitional phase between normal aging and Alzheimer's disease (AD) and is characterized by subtle cognitive deficits as well as structural changes in the brain. Volumetric analysis of gray matter (GM) and white matter (WM) using MRI provides crucial biomarkers for early diagnosis and monitoring of disease progression. This study investigated volumetric differences between healthy controls (HC) and MCI patients using advanced neuroimaging and AI‐assisted analysis techniques.

**Method:**

High‐resolution T1‐weighted MRI scans were used to quantify the volume changes of GM and WM in both groups. Automated segmentation and volumetric analysis were performed using the deep neural network Vb‐Net, which is optimized for precise quantification of brain structures. Boxplots were generated to visualize the regional volume distribution between HC and MCI. In addition, a k‐nearest neighbors (KNN) classifier was used to distinguish between HC and MCI based on volumetric features. Classification performance was evaluated using ROC curves.

**Result:**

MCI patients showed a significant reduction in GM volume in key regions of cognitive processing, particularly in the hippocampus, medial temporal lobe, and precuneus (*p* < 0.05). WM volume also showed significant decreases, particularly in frontal and temporal regions, suggesting early neurodegenerative changes. Boxplot analyses showed a clear separation of regional volume distributions between HC and MCI. The KNN classifier achieved high discrimination between HC and MCI, with a promising area under the ROC curve (AUC).

**Conclusion:**

Compared to other available AI tools, Vb‐Net has been adopted due to its outstanding ability to precisely segment and volumetric quantify brain structures. While traditional deep learning models such as U‐Net or VoxelMorph are designed for general image segmentation tasks, Vb‐Net is specifically developed for neuroanatomical analysis. Its deep residual and 3D feature extraction mechanisms enable higher accuracy in distinguishing small volume changes, which are crucial for early diagnosis of MCI.